# A Signal-Processing-Based Simulation System for High-End Stereo Headsets

**DOI:** 10.3390/s24072190

**Published:** 2024-03-29

**Authors:** Anna Zuccante, Alessandro Fiordelmondo, Pierluigi Bontempi, Sergio Canazza

**Affiliations:** 1 Centro di Sonologia Computazionale (CSC), Department of Information Engineering (DEI), University of Padua, Via Gradenigo 6/b, 35131 Padova, Italy; anna.zuccante@studenti.unipd.it (A.Z.); fiordelmondo@dei.unipd.it (A.F.); pierluigi.bontempi@phd.unipd.it (P.B.); 2 Department of Art History, Film Studies, Media Studies, and Music (DIUM), 33100 Udine, Italy

**Keywords:** headphones, digital signal processing, simulation system, headphones virtualization, binaural audio, headphones impulse responses

## Abstract

In recent years, headphones have become increasingly popular worldwide. There are numerous models on the market today, varying in technical characteristics and offering different listening experiences. This article presents an application for simulating the sound response of specific headphone models by physically wearing others. In the future, for example, this application could help to guide people who already own a pair of headphones during the decision-making process of purchasing a new headphone model. However, the potential fields of application are much broader. An in-depth study of digital signal processing was carried out with the implementation of a computational model. Prior to this, an analysis was performed on impulse response measurements of specific headphones, which allowed for a better understanding of the behavior of each set of headphones. Finally, an evaluation of the entire system was conducted through a listening test. The analysis of the results showed that the software works reasonably well in replicating the target headphones. We hope that this work will stimulate further efforts in the same direction.

## 1. Introduction

The past decade has seen a growing trend in the headphones market at a level that is expected to reach a value of USD 163.83 billion by 2030 [1]. Evidence suggests that the widespread diffusion of portable devices, such as smartphones, tablets, and music players, is among the most important factors for this trend. On the other hand, headphones are also a commonly used tool in professional settings, such as audio production and critical listening, where they are commonly alternated with listening on loudspeakers [2]. A specific field of application for headphones, particularly relevant today, is immersive audio, both from a professional and a personal point of view [3]. Given the spread of headphones in many aspects of people’s daily lives, users are increasingly demanding better performance and new features. Consequently, over the years, companies and researchers have sought to offer increasingly innovative products that could also encapsulate the strengths of other audio systems, often through digital audio signal processing (DSP) techniques. In this regard, three main areas of intervention can be identified: frequency response correction of specific headphones models—if not individual headphones [4]—headphone simulation of the listening experience achievable through the use of loudspeakers [5], and binaural encoding of immersive audio formats [6]. The relevance of these areas is also made evident by the fact that world-leading music production hardware and/or software manufacturers, including Waves (NX Virtual Studio), Steinberg (Immerse and Immerse Virtual Studio), Sonarworks (SoundID Reference), and Slate Digital (VSX), have offered dedicated solutions in recent years. In this paper, a different application is proposed, which, instead of trying to simulate different listening environments through headphones or correcting the frequency response of the headphones to obtain a virtually flat one, aims to simulate the auditory characteristics of headphones when the user is wearing another pair of headphones. The state of the art of such an application is rather limited. Among the most significant research, the work of Olive [7] should be mentioned, who describes a method to conduct virtual headphone listening tests and presents the results of a validation experiment in which listeners had to rank, in order of quality, listening with standard headphones without modification and with frequency response corrections. It is also worth mentioning the open-source software AutoEQ [8], which contains a tool to automatically equalize the frequency responses of headphones with the goal of obtaining a pleasant sound for the user. In the commercial arena, the VSX system produced by Slate [9] is proposed to simulate numerous headphone models but requires the use of a dedicated proprietary headphone. Since we propose to offer the possibility of simulating the sound response of other headphones through the use of any supported headphone models, we believe that our work has marked traits of originality and can represent a significant contribution to the field. The potential fields of application are numerous: buyer support in headphone selection, different simulated listening experiences in audio production, selection of the best-simulated model depending on the specific application, uniformity of sound signaling used for testing, experimentation, and professional applications in the presence of variable hardware. For example, the relevance of the frequency response of headphones (and their non-interchangeability) in medical applications has been highlighted in [10]. The impact of the specific headset on the subjective perception of the quality of customized head-related transfer functions (HRTFs) is described in [11]. HRTFs are crucial in binaural immersive audio [12], a field with an exponential growth in interest. Another field of application could be sound archives, where researchers are interested in improving and expanding the listening experience of audio documents and matching the most suitable virtual headphones for different sound materials without having to physically replace the device [13,14,15].

The work described here is currently being carried out in the Centro di Sonologia Computazionale (CSC) laboratory [16] at the Department of Information Engineering (DEI) of the University of Padua in collaboration with Audio Innova srl [17]. The project was born from the idea of creating a commercial application that allows the simulation of high-fidelity headphones so that a user can simulate and compare the sound of different models of headphones with the one they are wearing at the time of listening.

The structure of the paper is as follows. Section 2 provides an analysis of headphone measurements and their acquisition. Section 3 and Section 4 outline the materials and methods used for the development and testing of our software. In Section 5, the accuracy of the software is evaluated. Section 6 describes the experiment conducted with various participants to assess the system, and the results are presented in Section 7. Lastly, a final discussion of the work is provided in the concluding sections.

## 2. Headphone Measurements

Acoustic engineering relies on the impulse response (IR) technique as a crucial tool for estimating the acoustic parameters and characteristics of environments and sound systems. In order to obtain precise measurements of an IR, specific criteria need to be fulfilled [18]. First, the source should possess a well-defined and reproducible sound power radiation. An ideal acoustic pulse is described as an impulsive signal with an infinitely short duration and an infinitely high power and unit energy. At the same time, the receiver should exhibit high sensitivity in different directions and frequencies. The acoustic parameters derived from these measurements must be calculated using well-established algorithms and associated software implementations. Finally, since it is often useful to compare different measurements, it is advisable to keep the IRs for future consultation. To acquire an estimated impulse, one potential method involves using blank pistols, balloons, and other mechanical devices that can generate impulsive signals of varying frequencies at high levels. Nevertheless, these methods lack controllable direction and reliable reproducibility, which are essential for obtaining accurate measurements. Fortunately, it is feasible to obtain an IR by performing deconvolution on the response of an excitation signal, as long as the signal is clearly defined and has enough energy at each relevant frequency. Currently, the most frequently employed excitation signals are the maximum length sequence (MLS) and swept sine. The swept sine has an advantage over the MLS in that it uses a time-growing exponential frequency sweep to separate the linear and nonlinear responses of the room. This behavior is crucial for the conditions of our project (see Section 4).

To measure the IR of headphones, special techniques and sensors are necessary. Typically, two approaches can be used: the microphone in real ear (MIRE) technique and the usage of an acoustic test fixture (ATF). The former method entails placing a probe microphone at a minimum distance of 4 mm from the entrance of the ear canal of a person to assess the IR [19]. However, this technique is not widely employed, primarily due to the challenges involved in taking measurements within the ear canal. First, the sensors inserted into the ears of the subjects could be uncomfortable and move during measurements, resulting in changes during testing. Furthermore, the number of test subjects affects the accuracy. The study in [20] showed that depending on the subject wearing the in-ear microphones, moderate fluctuations in low-frequency responses and significant resonances at high frequencies can occur. In contrast, the second method eliminates the requirement for a human subject by employing an ATF [21]. This instrument replicates the acoustic impedance of specific body regions, specifically the ears, to faithfully replicate the acoustic pathway of sound from the headphones to the eardrum. ATFs are extensively utilized in the field of acoustics due to their ability to resolve numerous issues associated with the previous approach, such as the reproducibility of experiments and the ability to compare results obtained using different ATFs but following the same standard. The ATF presents unique features. Firstly, it integrates measurement microphones that have the ability to capture acoustic signals produced by headphones. These sensors are placed within ear simulators, which are small cavities designed to mimic the impedance of the human ear. Some ATFs also replicate the structure of the outer ear, including the pinna, concha, and ear canal. The overall setup also requires the use of a headphone amplifier, a microphone preamplifier, and a digital-to-analogue converter (DAC). All these components are generally included in the audio interface used. In addition, software is needed to capture the IR and organize the data properly; a good example is the free Room Equalization Wizard (REW) software [22]. The acquisition of headphone IRs is a crucial step for comprehending their properties and improving DSP algorithms. As open science gains more recognition, researchers often turn to online datasets to access a variety of standardized data. With this in mind, we conducted extensive research and carefully selected datasets. Our selection criteria included the use of ATF devices, the relevance of the datasets in the industry and the international hearing community, and the possibility of comparing them. By “comparable”, we refer to the utilization of similar measurement devices following the specifications of the IEC 60318-4 [23] and IEC 60318-7 [24] standards for the measuring rig. Additionally, we specifically sought out IR measurements taken using a logarithmic sweep signal. After all these evaluations, we found the following datasets: Crinacle [25]; the measurements provided by Oratory1990, which are maintained by Jaakko Pasanen in the AutoEQ database [26]; Innerfidelity [27]; Rtings [28]; SoundStage! Solo [29]; and Headphone Test Lab [30]. The initial dataset was acquired using GRAS KB5000 [31]/KB5001 [32] artificial pinnae and two GRAS RA0402 [33] pre-polarized high-resolution IEC 60318-4 ear couplers. The second dataset was measured with GRAS KB5000/KB5001 artificial pinnae and a GRAS45BC-10 KEMAR [34]. The Innerfidelity dataset was measured using the Head Acoustics HMSII.3 head acoustics simulator [35]. The Rtings dataset also utilized the same type of head acoustics simulator. SoundStage Solo primarily used a GRAS 43AG ear/cheek simulator [36] GRAS RA0402 ear simulator with GRAS KB5000/KB5001 simulated pinnae. Lastly, Headphone Test Lab employed various types of ATFs, which can be found listed here: https://headphonetestlab.co.uk/test-details-hardware-and-software-listing (accessed on 13 March 2024).

## 3. Materials

The simulator was coded using Python 3.8 exclusively. To test the software, a set of circumaural headphones with dynamic drivers was chosen (Table 1).

The selected headphones were differentiated by their acoustic design and included closed, semi-open, and open models. The decision to use this specific type of headphones was based on the preferences of audiophiles. One factor contributing to this is the inherent naturalness of sound perception in everyday life. Aside from the reflections and diffractions that sound undergoes in space, the human perception of sound is also naturally influenced by reflections from the body itself, such as the torso, ears, and head, before reaching the eardrum [37]. Unlike loudspeakers, headphones bypass this phenomenon by directly coupling with the ear, resulting in a less natural perception of sound [38]. However, among all types of headphones, circumaural headphones come closest to replicating the sound perceived from a loudspeaker. This is primarily due to their design and shape, which aim to preserve these distortions as much as possible.

Moreover, we aimed to choose one dataset from the headphone measurement study (see Section 2) to assess the robustness of our system. By utilizing datasets obtained from different contributors and contexts, researchers can reduce the biases that are present in proprietary datasets and generate more robust and widely applicable findings. This approach enhances the overall quality and relevance of scientific research outcomes. Specifically, we tested our software using the datasets acquired from Crinacle and the measurements provided by Oratory1990.

From an operational point of view, the developed software is based on a graphic interface (GUI). Specifically, the user is prompted to select the monitor and target headphones, then the audio they wish to filter, and finally the folder in which all results are saved (graphs, filter IR, and filtered audio).

## 4. Methods

**Figure 1:**
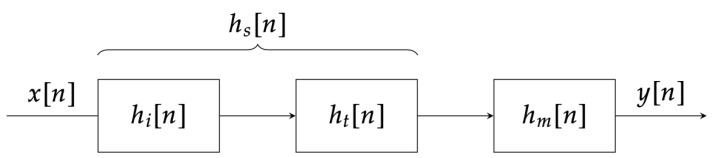
Pre-filtering of monitor headphones. The impulse responses of the target, denoted as $h_{t}\left[n\right]$, and the monitor, denoted as $h_{m}\left[n\right]$, were practically determined following the procedure outlined in Section 2. Specifically, the software underwent testing using measurements from Crinacle and Oratory1990. The impulse responses of the headphones were captured by employing a swept sine excitation signal. Furthermore, for the Crinacle dataset, artificial pinnae GRAS 135 KB5000/KB5001 and two pre-polarized high-resolution 136 IEC603318-4 ear couplers from GRAS RA0402 were utilized. In the case of the Oratory1990 dataset, artificial pinnae GRAS KB5000/KB5001 and a KEMAR GRAS45BC-10 were employed.

As mentioned in the Introduction, the proposed application aims to create an environment where the listener can ideally replicate the same listening experience they would have in real life while using the headphones in question. The term “ideally” is used because the application cannot replicate the physical characteristics of headphones, such as their weight or how they fit around the listener’s ear. In other words, the goal is to provide a tool that can assist a user in choosing a new pair of headphones or that can give them a reasonable approximation of the listening experience they would have on another headphone model while being aware of the inherent limitations of software emulation. For the rest of this article, we will use the term target to refer to the headphones we aim to simulate using another pair of headphones, which we will call a monitor. The idea behind the functioning of the simulator involves the application of a filter that simultaneously eliminates the contribution of the monitor headphones and adds that of the target headphones, The model can be described using the block diagram shown in Figure 1.

In this diagram, the simulation is conducted by incorporating a specific filter $h_{s}\left[n\right]$. This filter is formed by combining the inverse filter of the impulse response of the monitor, $h_{i}\left[n\right]$, with the impulse response of the target, $h_{t}\left[n\right]$. The resulting filter is then inserted before the impulse response of the monitor, $h_{m}\left[n\right]$.

The equivalent equation in the frequency domain is the following:

(1) $$\begin{array}{cc} Y(f) & =H_{s}\left(f\right)\cdot H_{m}\left(f\right)\cdot X\left(f\right)\\ \; & =H_{t}\left(f\right)\cdot H_{i}\left(f\right)\cdot H_{m}\left(f\right)\cdot X\left(f\right)\\ \; & =H_{t}\left(f\right)\cdot \frac{1}{H_{m}\left(f\right)}\cdot H_{m}\left(f\right)\cdot X\left(f\right)\\ \; & =H_{t}\left(f\right)\cdot X\left(f\right) \end{array}$$

Through this procedure, after some simplifications, it is possible to obtain the desired scenario of the sonic response of the target headphones. In order to implement this procedure, it is fundamental to assume that headphones can be considered linear- and time-invariant (LTI) systems with respect to the audio signal given as input. If this assumption can be made, then theoretically it is possible to simulate the auditory characteristics of any headphones only by replicating their transfer function. However, when a loudspeaker, headphones, or any other transducer-based system is exposed to high vibration levels, particularly in the electro-mechanical transducer, it experiences nonlinear phenomena [39]. These phenomena result in distortion effects in the sound reproduced, which subsequently makes it difficult to accurately model the system. Nonetheless, if it was feasible to remove these distortions from the measured IRs, the original model would still be applicable, since the IRs in the pipeline would only be relative to the linear component of the respective system. This scenario occurs when an exponential sinusoidal sweep method is used during the measurement process [40]. This approach allows for the distinction between the linear and nonlinear components of the system by leveraging the characteristics of the excitation signal. Consequently, a straightforward windowing process can be employed to extrapolate only the linear portion of the response. By taking advantage of measurements obtained with this type of approach, it is then possible to apply the model initially described, since, under these conditions, it is considered valid.

**Figure 2:**
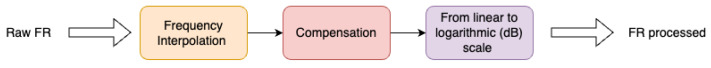
Preprocessing of the frequency responses.

The simulator workflow includes two main tasks: pre-processing the frequency responses of the monitor and target headphones and creating and implementing the filter. Figure 2 shows the series of operations performed on the frequency responses in the first task to optimize the filter development.

Interpolation is a crucial initial step in analyzing frequency responses using measurements from external sources with limited information. Audio signals can be recorded or processed at different sampling frequencies. When comparing or combining signals with different sampling frequencies, interpolation can be used to adjust the sampling frequency and align the signals for accurate comparison or further processing. Interpolation ensures that signals with different sample rates or time points are aligned, avoiding anomalies during analysis. To ensure meaningful comparisons, it is essential to apply the same interpolation techniques to both target and reference headphones. The use of different methods can cause misalignment that may hinder accurate evaluations. To ensure a fair and accurate assessment of signal similarities or differences, it is recommended to maintain consistency in the interpolation steps when comparing signals. The CubicSpline interpolator was chosen for its high effectiveness, reliability, and computational efficiency [41].

Subsequently, a compensation operation is implemented to remove the influence of the measurement device, which is inevitably introduced into the impulsive responses during the measurement phase. We examined various compensation curves, including the diffuse field, free field, and the Harman AE OE 2018 Target [42].

Finally, transitioning from linear to a logarithmic scale has been implemented to significantly simplify signal processing. For instance, when convolving the monitor’s inverse response with the target, which simplifies to multiplication in the frequency domain, preserving the logarithmic scale enables direct addition between the monitor’s inverse frequency response and the target’s frequency response. Furthermore, the use of a logarithmic scale in audio applications is well aligned with the perceptual characteristics of human hearing. This facilitates a more intuitive and efficient representation of various audio signal attributes, such as the loudness, frequency, and dynamic range. It is important to note that when transitioning back to the time domain to obtain the final impulse response, the inverse fast Fourier transform (iFFT) is applied to the linear scale of the frequency response curve. Thus, in this final step, we use a linear scale instead of a logarithmic one.

**Figure 3:**
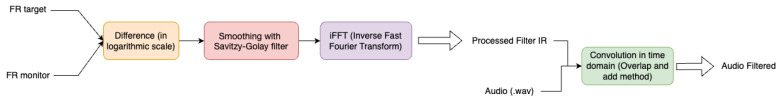
Development and application of the filter.

Once the preprocessing is completed, the simulator initiates its core functionality (Figure 3).

The initial step involves the development of filters using convolution, following the model described earlier. Once the first version of the filter is obtained, an operation of smoothing is applied to reduce the effects of noise on the IR, preserving only the perceptually relevant temporal characteristics of the original response at the same time. Among the different types of smoothing filters, the Savitzky–Golay filter was chosen. This choice is justified by the filter’s tendency to better preserve the shape of the curve (especially the height and width of the peaks), removing as much noise as possible from the corrupted signal [43,44]. Since the noise is greater at high frequencies, it was decided to construct a smoothing filter based on the application of a stronger filter on the treble frequencies and a softer filter for the low–mid frequencies. In this way, we can perform smoothing in greater detail on the most corrupted frequencies. Ultimately, the application achieves the desired simulation by convolving the newly processed filter with an initially selected audio. Convolution is performed using the overlap and add method in the time domain. This method is an efficient way to calculate the discrete convolution of a lengthy signal with a short IR. After the convolution is computed, a volume normalization step, exploiting the root mean square (RMS), is applied to ensure that the resulting audio file has the same volume as the original input audio. This ensures that there is minimal influence during the listening tests.

## 5. Simulation Accuracy

**Figure 4:**
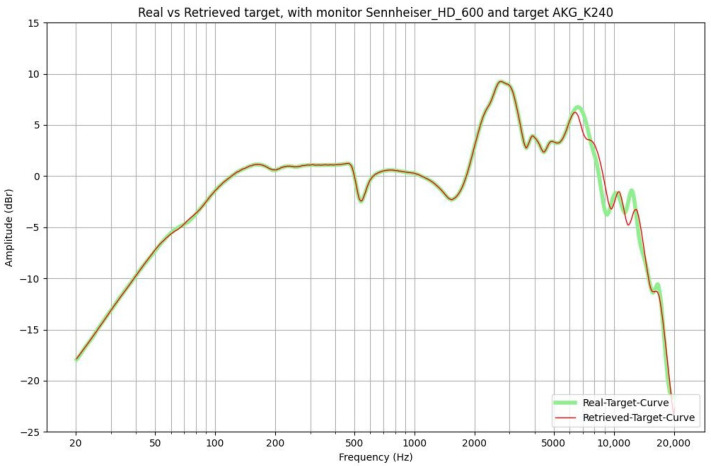
Let Sennheiser HD600 be the monitor headphones and AKG K240 MKII be the target headphones. Here, the real target and the retrieved target are displayed.

**Figure 5:**
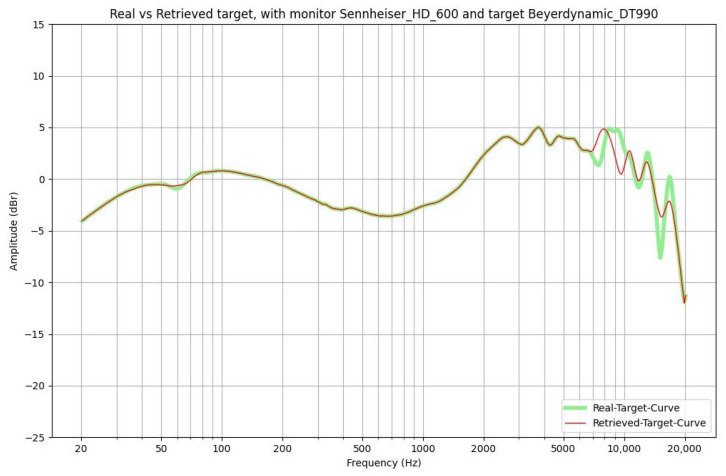
Let Sennheiser HD600 be the monitor headphones and Beyerdynamic DT990 be the target headphones. Here, the real target and the retrieved target are displayed.

The first feedback on the functionality of the application is presented through some graphs, such as the one visible in Figure 4 and Figure 5.

The results are plotted in the audible frequency range of 20 Hz to 20 kHz and are derived from a function implemented in the software. The following curves are shown: in light green, the frequency response of the target headphones, and in red, the derived curve if we apply the filter to the monitor headphones. The graphs demonstrate that the system is working reasonably well since the two curves nearly coincide. The only noticeable discrepancies occur in the higher frequency range and can be attributed to the smoothing process discussed above. To provide a more quantitative evaluation of the accuracy of the simulation, we computed the cross-correlation function between the real frequency response of the target headphones and the one obtained from the monitor using the filter derived from the model. The outcome is a numerical value ranging from 0 to 1, where a value closer to 1 indicates a better simulation. In our study, we used Kendall’s tau correlation coefficient. The highest value, 0.98, is achieved when using AKG K240 MKII as the monitor and Beyerdynamic DT770 as the target. On the contrary, the lowest value, 0.67, is obtained when using Beyerdynamic T1 as the monitor and Beyerdynamic DT990 as the target. The average value across all combinations is 0.81, indicating a satisfactory accuracy. As a result, we felt confident in conducting listening tests.

## 6. Assessment

**Figure 6:**
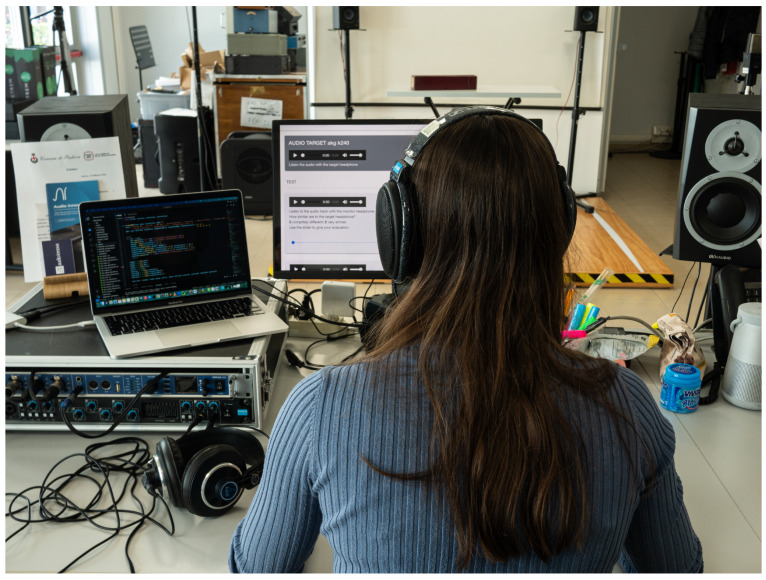
A participant wearing the Sennheiser HD600 (monitor headphones) during the evaluation phase with the AKG K240 MKII, the target headphones to be simulated, on the side.

The listening test was conducted at the CSC laboratory [45] on a sample of people with different levels of expertise in the field. In Figure 6, a participant is shown taking part in our experiment. Before starting the experiment, a brief survey was conducted. The survey was designed to collect crucial information about the age of the participants, their listening experience, and potential auditory problems. To assess their listening experience, participants were asked to rate their level on a scale from “Unfamiliar” to “Expert”, supplemented by a brief description of their experience and the system they used the most. The combination of qualitative and quantitative data will contribute to a comprehensive understanding of the participants and ensure a nuanced analysis of the results of the listening test. Once the participant finished the brief questionnaire, they were granted access to the listening text. The experiment was divided into three trials, each focusing on a different audio stimulus: white noise, a segment from a rock song, and a segment from a classical song. Each trial was replicated for each pair of audio headphones, resulting in a total of six simulations. The participant was provided with five audio tracks for each stimulus. The first track has to be listened to using the target headphones, while the subsequent four tracks have to be listened to using the monitor headphones. The last four tracks are filtered versions of the original track, and the participant was required to rate their similarity to the reference audio track. Among the last four tracks, one is considered the correct response, and we expect that the participant will give it the highest score. This track is the audio filtered by our system for the target we are simulating. Another track is unfiltered. The remaining two tracks represent the same track but are filtered for a different target than the one we want to simulate. Since these two stimuli are equal, they are considered control options, with the expectation that the participant will assign them the same score to ensure that the responses are not random and that the participant possesses adequate critical listening skills. All of these tracks, except for the first one, were always presented in a randomized order to minimize any influence on the participant. The audio playback could be repeated as many times as needed. Each participant’s total test duration should be around 15–20 min. For this experiment, the Sennheiser HD600 was selected as the monitor, while the AKG K240 MKII and Beyerdynamic DT770 80Ω were chosen as the target headphones.

## 7. Results

Seventeen individuals, with an average age of 27, were voluntarily recruited as participants. The youngest participant was 22 years old, while the oldest was 39 years old. The objective was to identify participants who, on average, were more successful in recognizing the hidden reference (the audio track filtered by our system for the correct target). To achieve this, we initially calculated the algebraic difference between the rating given to each incorrect track and the rating given to the hidden reference. Subsequently, for further statistical analysis, a one-sided T-test was performed on these algebraic differences to ensure that their mean significantly deviates from zero. The result of this test was the *p*-value, which indicates the probability that the mean of a set of values is equal to a population mean (in this case, 0). If the *p*-value is below 0.05, the probability of the two means being equal is very low. Hence, for participants meeting this criterion, we could conclude that they were capable of distinguishing the hidden reference from the other tracks. Upon careful examination of the obtained results, it was discovered that the selected group of participants comprises individuals who possess the ability to accurately identify the target and also assign equal value to the control tracks.

**Figure 7:**
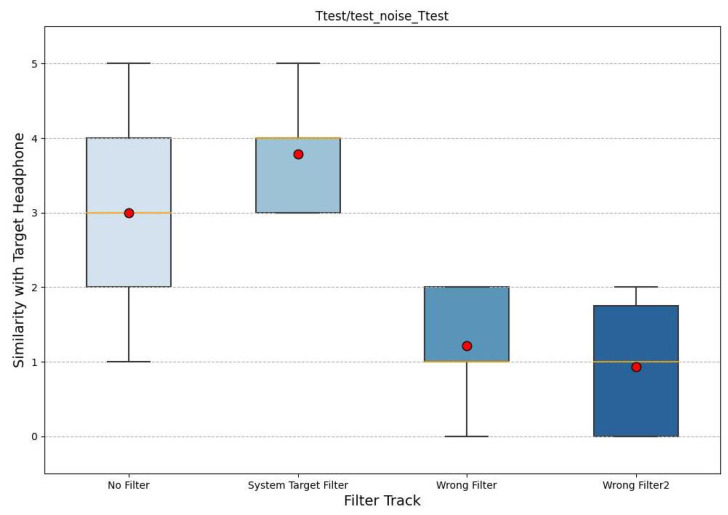
The box plot of the similarity evaluation given by the participants for the white noise stimuli. On the x-axis are the four tracks and on the y-axis is the similarity value.

**Figure 8:**
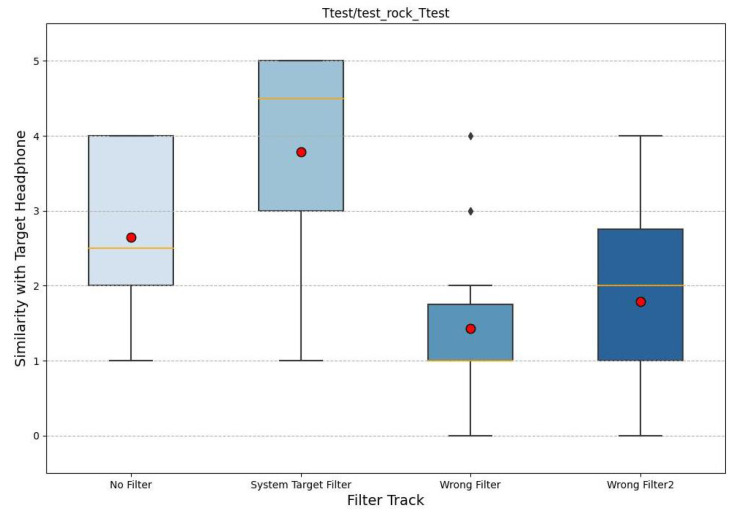
The box plot of the similarity evaluation given by the participants for the rock music stimuli. On the x-axis are the four tracks and on the y-axis is the similarity value.

**Figure 9:**
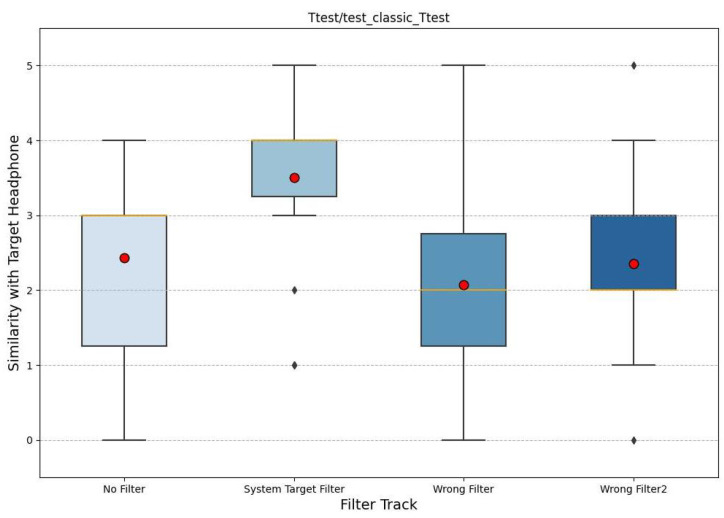
The box plot of the similarity evaluation given by the participants for the classical music stimuli. On the x-axis are the four tracks and on the y-axis is the similarity value.

We opted for box plots as they are better able to summarize the statistics of a dataset. In particular, in the figures, we note the median in yellow; the box plots were constructed according to the quartile criterion. The total length is called the interquartile range (IQR) and, like the standard deviation, it represents a measure of the variance of the data. Outside the boxes, the whiskers cover the data that fall outside the boxes, with a maximum extension of 1.5 ∗ *IQR*. All data not reached by the whiskers are indicated by a dot and are called outliers. Also, in these graphs, the mean is plotted with a red circle. The following graphs (noise in Figure 7, rock in Figure 8, and classical in Figure 9) represent the results, where the x-axis shows the four filtered tracks that the participant listens to with the monitor headphones, while the y-axis shows the range of similarity ratings with the target headphones.

Considering all three graphs together, it is possible to note a higher mean for the audio with the target filter, which tends to deviate more from all others. Also, the control tracks are significantly lower. When comparing the three graphs separately, it can be seen that white noise is the audio in which the participants perform better. In fact, the variations in the results are smaller, and the average difference between the audio with the target filter and the other tracks is greater. In contrast, the classical music excerpt is the audio track where there is more uncertainty, such that the averages of the ratings are almost equal and the unfiltered track has a higher average similarity than the audio with the target filter.

Furthermore, following a previous experiment [46], a further evaluation was performed using Tukey’s statistical method and the honestly significant difference (HSD). This tool allows pairwise comparisons between more than two variables and is usually used to find averages that are significantly different from each other. In our case, it was applied to compare the ratings of the four filtered tracks. Tukey’s HSD method returns several values, including the *p*-value (which we had measured during the extraction of the expert subgroup). The results of impulse white noise (the most significant) are shown in Table 2.

In particular, we are interested in the *p*-values of the “System Target Filter” row, which, if they are less than 0.05 (in bold), indicate that the two groups considered are statistically different. The values within the table further confirm the division into two clusters; in fact, “No Filter” and “System Target Filter” are statistically different from the two control tracks defined by “Wrong Filter” and “Wrong Filter2”. In the same table, we can also observe a near equality between the two control traces; in fact, the *p*-value between the two is very close to 1.

## 8. Discussion

The findings demonstrate that the system effectively replicates the characteristics of one set of headphones in another, even for audio that differs in frequency, time, and sound. In particular, from the box plots of noise and rock, we can identify the creation of two clusters: one consisting of the stimulus filtered with our system for the correct target and the stimulus without any filter applied, and the other group consisting of the two tracks filtered by our system for a different target. The first group obtained a higher similarity value on average than the other, with the stimulus filtered by our system obtaining the highest value. The high value given even to the unfiltered track probably indicates the difficulty in isolating the physical aspects of the headphones worn during the evaluation. Indeed, one of the limitations of our system is the impossibility of simulating the physical components of the headphones, from the drivers to the pads, as these are hardware components and not software. This impossibility led us to limit ourselves to headphones belonging to the same typology, specifically high-end dynamic over-ear headphones (as indicated in Section 3). However, in order to assess how well the system, despite its limitations, can simulate a given set of headphones, it would be interesting to verify this through on-site measurements of the headphones themselves with and without the implemented system. In this way, from the derived headphone curves, we can evaluate the differences in order to assess how much the hardware can influence the frequency response alone.

Furthermore, an interesting observation emerging from the results is that experts tend to have more self-reported experience (refer to Table 3) compared to all participants, suggesting that they are more at ease in the listening field.

Notably, when examining their responses regarding musical experience, electronic music composers and individuals knowledgeable in music production stand out. On the other hand, participants outside this group of experts predominantly identified themselves as amateur music listeners, often not owning high-quality listening devices. This remark aligns with the aforementioned results, indicating that involving experienced individuals can minimize the likelihood of random and hasty responses from participants. To sum up, the findings presented here provide an understanding of the average behavior of our system during simulations of a specific type of headphone.

## 9. Conclusions

In this contribution, we presented a system capable of replicating the listening experience one would have on one model of headphones (target) using another model of headphones (monitor). The results obtained through the listening tests performed were encouraging, particularly in relation to the participants with more experience in the field of professional audio. The long-term goal of this research is to collect an IR dataset and to build associated software able to replicate the timbre characteristics of popular headphones on other headphones models with an even higher level of accuracy. This will facilitate comparisons between different models and offer alternative listening experiences to audiophiles and generally to people working professionally in the audio field without the need to change headphones.

## Figures and Tables

**Table 1:** List of Hi-Fi headphones used in the simulation.

Headphone Name	Acoustic Design
AKG K240 MKII	Semi-Open
AKG K701	Open
Beyerdynamic DT770 M 80Ω	Closed
Beyerdynamic DT990 250Ω (with earpads)	Open
Beyerdynamic T1	Open
Focal Stellia	Closed
Sennheiser HD600	Open
Sennheiser HD650	Open
Sennheiser HD800S	Open
Shure SRH1540	Closed

**Table 2:** *p*-values of “expert participants” on noise audio computed using Tukey’s HSD method in order to determine whether the differences are statistically significant. The values in bold indicate *p*-values lower than 0.05, which indicate a significant statistical difference.

Noise	No Filter	System Target Filter	Wrong Filter	Wrong Filter2
No Filter	-	1.98 × 10^−1^	**1.76 × 10^−4^**	**1.41 × 10^−5^**
System Target Filter	1.98 × 10^−1^	-	**1.39 × 10^−7^**	**9.53 × 10^−9^**
Wrong Filter	**1.76 × 10^−4^**	**1.39 × 10^−7^**	-	8.84 × 10^−1^
Wrong Filter2	**1.41 × 10^−5^**	**9.53 × 10^−9^**	8.84 × 10^−1^	-

**Table 3:** List of some statistics values of the acoustic tests. “Expert participants” identifies the group of experts resulting from the post-screening.

	All Participants	Expert Participants
Average Age	27.29	28.71
Average Experience	2.35	3.57

## Data Availability

Dataset available on request from the authors.

## Abbreviations

The following abbreviations are used in this manuscript:

DSP	Digital Signal Processing
HRTF	Head-Related Transfer Function
IR	Impulse Response
MLS	Maximum Length Sequence
ATF	Acoustic Test Fixture
IQR	Interquartile Range
HSD	Honestly Significant Difference
